# Untargeted lipidomics and metagenomics reveal the mechanism of aspirin eugenol ester relieving hyperlipidemia in ApoE−/− mice

**DOI:** 10.3389/fnut.2022.1030528

**Published:** 2022-12-19

**Authors:** Xiao-Rong Lu, Xi-Wang Liu, Shi-Hong Li, Zhe Qin, Li-Xia Bai, Wen-Bo Ge, Jian-Yong Li, Ya-Jun Yang

**Affiliations:** Key Laboratory of New Animal Drug Project of Gansu Province, Key Laboratory of Veterinary Pharmaceutical Development of Ministry of Agriculture and Rural Affairs, Lanzhou Institute of Husbandry and Pharmaceutical Science of Chinese Academy of Agricultural Sciences, Lanzhou, China

**Keywords:** aspirin eugenol ester, hyperlipidemia, lipidomics, ApoE-/- mice, metagenomics

## Abstract

Hyperlipidemia is induced by abnormal lipid metabolism, which can cause the occurrence of cardiovascular diseases and lead to grievous injury to health. Studies showed that AEE had a significant therapeutic effect on hyperlipidemia and is likely to be associated with the up-regulation of cholesterol 7-alpha hydroxylase (CYP7A1), the key enzyme for cholesterol conversion to bile acids, but no research confirmed whether the effect of AEE on hyperlipidemia was related to the gut microbiota and liver lipids. At the same time, more and more studies have shown that gut microbiota and lipids are closely related to hyperlipidemia. Hence, in this study, we investigated the effects of AEE on liver lipids through LC-MS-based untargeted lipidomics and the effects of AEE on gut microbiota based on cecal contents metagenomics by Illumina sequencing in HFD-induced hyperlipidemia ApoE−/− mice at the overall level. The results of lipidomics showed that AEE relieved hyperlipidemia by decreasing the concentration of 10 PEs and 12 SMs in the liver and regulating the pathways of glycerophospholipid metabolic pathway, sphingolipid signaling pathway, and NF-kB signaling pathway. The results of metagenomics concluded that AEE treatment changed the composition of gut microbiota and regulated the functions of lipid transport and metabolism, as well as the metabolism of bile acids and secondary bile acids. The results of the joint analysis between lipidomics and metagenomics showed that the abundance of Verrucomicrobia, Verrucomicrobiales, Candidatus_Gastranaerophilales, and Candidatus_Melainabacteria was significantly positively correlated with the concentration of SM (d18:1/18:0) and PE (16:0/18:1) in the process of AEE alleviating hyperlipidemia in mice. In conclusion, these results suggested that the effect of AEE on hyperlipidemia was closely related to the gut microbiota by the change of bile acids and liver lipids.

## Introduction

Hyperlipidemia is caused by abnormal lipid metabolism and mainly manifests as changes in one or more indicators of serum lipids including triglycerides (TG), total cholesterol (TCH), low density lipoprotein cholesterol (LDL), and high density lipoprotein cholesterol (HDL) (1). Hyperlipidemia is closely related to unhealthy eating habits and lifestyle. In the body, lipids are important components required for performing various homeostatic, physiologic actions and are the essential substances for basic cellular metabolism. The lipid substances in plasma are collectively referred to as blood lipids. Blood lipid metabolism is very active, and blood lipid level can reflect the body lipid metabolism (2). Cardiovascular and cerebrovascular diseases caused by hyperlipidemia from lipid metabolism disorder are the leading cause of death in the world, with high incidence and heavy burden (3, 4). Therefore, research on these diseases has attracted more and more attention all over the world. Hyperlipidemia causes an increasing number of cardiovascular diseases in companion animals such as dogs and cats. It has been reported that hyperlipidemia has been found in a variety of breeds of dogs, such as Miniature Schnauzers, Shetland Sheepdogs, Beagles, and other breeds. Companion animals are asymptomatic in the pre-hyperlipidemia phase, so preventive drugs for hyperlipidemia are needed urgently (5, 6). Therefore, there is an urgent need for pet-specific drugs for the treatment of hyperlipidemia.

Aspirin eugenol ester (AEE) is a new pharmaceutical compound synthesized by esterification of aspirin and eugenol based on the prodrug principle. Previous studies showed that AEE had the effects of anti-inflammation, antipyresis, analgesia, blood lipid reduction, anti-oxidation, and prevention of atherosclerosis and thrombosis, and the effect was superior to that of the prodrugs Asp and/or Eug (7–9). AEE mainly played a role in lowering blood lipids by improving the disordered metabolic profile and the disordered intestinal flora, and promoting the conversion of cholesterol in the liver into bile acids and their excretion in the body, thereby improving hyperlipidemia (10). However, the molecular regulatory mechanism of AEE in reducing blood lipid is still not clear enough. No research confirmed whether the effect of AEE on hyperlipidemia is related to the gut microbiota and liver lipids.

Lipidomics is a new omics strategy to study lipid metabolism by determining lipid composition and identifying lipid biomarkers at the molecular level. Currently, through comprehensive and systematic qualitative and quantitative analysis of lipids, we can determine the metabolic pathways and functions of lipids as signal molecules to clarify the pathogenesis of the disease and the regulatory mechanism after drug intervention (11, 12). Recently, lipidomics has been used to study various diseases caused by abnormal lipid metabolism, such as atherosclerosis, non-alcoholic fatty liver disease (NAFLD), hyperlipidemia, fatty liver, hypertension, and so on (13). Gut microbiome is also known as “another organ of the body.” Numerous studies indicate that gut microbiome is closely related to a variety of diseases and the exertion of drug action, such as hyperlipidemia, NAFLD, atherosclerosis, etc. (14). Metagenomics is an important means of studying gut microbiome, elucidating their community structure, species classification, genetic functions, and metabolic pathways, etc. (15, 16). The combination of lipidomics and metagenomics has been used to investigate the mechanisms of many diseases and drugs (17). In the liver, bile acid synthesis is the main pathway for eliminating cholesterol. Bile acids play an important role in lipid metabolism and have a close interaction with gut microbiome (18).

In this study, basing on lipidomics by performing LC-MS analysis and metagenomics by Illumina sequencing, we explored the effects of AEE on hepatic lipid metabolism and gut microbiota in hyperlipidemia mice at the overall level. Bioinformatics methods were used to analyze the lipidomics and metagenomics data in order to explore the mechanism of AEE in the regulation of hyperlipidemia and provide the basis for the action mechanism and target discovery of AEE.

## Materials and methods

### Chemicals and reagents

Transparent crystal AEE with purity of 99.5% was prepared in Key Lab of New Animal Drug Project of Gansu Province, Key Lab of Veterinary Pharmaceutical Development of Ministry of Agriculture and Rural Affairs, Lanzhou Institute of Husbandry and Pharmaceutical Science of Chinese Academy of Agricultural Sciences. Carboxymethylcellulose sodium (CMC-Na) was supplied by Tianjin Chemical Reagent Company (Tianjin, China). Isopropanol, acetonitrile, and methanol were purchased from Thermo Fisher (A451-4, A998-4 and A452-4, Thermo Fisher). Chloroform was supplied by Greagent (G75915B, Greagent). GCA-^13^C was supplied by Isoreag Company (IR-30935, Shanghai, China). CDCA-D_4_ was supplied by Sigma Aldrich Company (614122, Sigma, MO, USA). CA-D_4_ and BHT were purchased from Yuanye company (S22155, B25909, Shanghai, China). Formic acid (FA) was supplied by Sigma Aldrich Company (64186, Sigma, MO, USA). Ammonium forma was purchased from Supelco Company (540692, Supelco, PA, USA). ND (12.3% lipids, 63.3% carbohydrates, and 24.4% proteins) was purchased from Keao Xieli Feed Co., Ltd. (Beijing, China) and the high fat diet (HFD) (40% lipids, 43% carbohydrates, and 17% proteins) was supplied by Research Diet. The TCH and LDL-C kits for serum were provided by Ningbo Medical System Biotechnology Co., Ltd. (Ningbo, China). Erba XL-640 analyzer (German) was used to measure blood lipid levels. MagPure Soil DNA KF Kit is used to extract DNA from the contents of the cecum. TruSeq Nano DNA LT Sample Preparation Kit (Illumina) for library construction. KAPA Library Quantification Kits (Kapa Biosystems) for library quality inspection.

### Animal experiment and study design

C57BL/6J male mice and ApoE−/− male mice aged 6–8 weeks and weighted 18–22 g were purchased from Gempharmatech Co., Ltd. (Nanjing, China). The mice were housed at laboratory with a 12 h light/dark cycle at 18–22°C and 48 ± 10% humidity, and acclimated for 1 week before the study beginning. The protocols and procedures for the animal study were approved by the Institutional Animal Care and Use Committee of Lanzhou Institute of Husbandry and Pharmaceutical Science of Chinese Academy of Agricultural Sciences (Approval No. NKMYD202108; Approval Date: 20 May 2021). Animal welfare and experimental procedures were performed strictly in accordance with the Guidelines for the Care and Use of Laboratory Animals issued by the US National Institutes of Health.

Six C57BL/6J mice were the ND (normal diet) group, in which mice were received normal diet (ND) for 10 weeks. Twenty-four ApoE−/− mice were randomly divided into four groups: in the HFD group, mice were received HFD for 10 weeks (*n* = 6); in the AEE H group, mice were received HFD and administrated with AEE (217 mg/kg/d body weight) for 10 weeks (*n* = 6); in the AEE M group, mice were received HFD and administrated with AEE (168 mg/kg/d body weight) for 10 weeks (*n* = 6); and in the AEE L group, mice were received HFD and administrated with AEE (118 mg/kg/d body weight) for 10 weeks (*n* = 6). After the last AEE gavage, mice were fasted up to 10–12 h and then mice were decapitated under deep anesthesia with ethyl ether. After that, the samples were harvested for further experiment. Firstly, blood was collected from the orbital vein for blood lipids analysis. Secondly, liver tissue samples were collected for lipidomics. Finally, the cecum was found in the abdominal cavity of the mouse. The cecum was cut with sterile scissors, placed in a petri dish and quickly transferred to the super-clean worktable. The cecum was cut open and the contents were all taken out to put into a sampling tube. Afterward, it was put into liquid nitrogen for quick-frozen and stored at −80°C. Both HFD induction and AEE administration were carried out simultaneously. AEE suspensions were prepared in 0.5% CMC-Na, and the mice in ND and HFD groups were received equal volume of 0.5% CMC-Na as AEE M treatment group. The ND, HFD, and AEE groups were selected for the studies of mice liver lipidomics and cecal contents metagenomic. Based on the effect of AEE on serum lipid, the AEE L group is designated as the AEE group.

### Liver lipidomics analysis

#### Liver sample pretreatment method

Each mouse liver sample was weighed 30 mg to centrifuge tube, and 300 μL methanol-water (1:1, V/V, containing GCA-^13^C, CDCA-D_4_, and CA-D_4_) and two small steel balls were added. Samples were placed at −20°C for 2 min and added to a grinder (60 Hz, 2 min). Then 300 μL chloroform was added into the mixture, vortexed for 30 s, extracted by ultrasonic method for 10 min, and allowed to stand at −20°C for 20 min. The mixture was centrifuged for 10 min (13,000 rpm, 4°C) and 200 μL of the lower chloroform layer was loaded into an LC-MS injection vial. After the lower layer solution was taken out, 300 μL chloroform-methanol (2:1, V/V) (containing 0.1 mM BHT) was continuously added into the centrifuge tube, vortexed for 30 s, and ultrasonic extraction was performed in an ice-water bath for 10 min. The mixture was allowed to stand at −20°C for 20 min, and then centrifuged for 10 min (13,000 rpm, 4°C). 300 μL lower chloroform layer in the centrifuge tube was continuously put into the original LC-MS vial for volatilization. After volatilization, the lipid residue in the LC-MS vial was reconstituted with 300 μL of 2-propanol-methanol (1:1, V/V) (vortexed for 30 s and sonicated for 3 min), 20 μL of the mixed isotope internal standard was added, and the solution was transferred to a 1.5 mL centrifuge tube. After the solution in the centrifuge tube was allowed to stand at −20°C for 2 h, the mixture was centrifuged for 10 min (13,000 rpm, 4°C) and 150 μL of the supernatant was loaded into an LC-MS injection vial with an internal cannula for LC-MS analysis. Quality control samples (QC) were prepared by mixing equal volumes of extracts from all samples.

#### Analysis of liquid chromatography and mass spectrometry

Lipidomics analysis was performed on liquid chromatography coupled with a tandem mass spectrometer system consisted of an AB Sciex Exion LC system (AB Sciex Corp, MA, USA) and an AB Sciex Qtrap 6500 plus mass spectrometer (AB Sciex Corp, MA, USA).

##### Chromatographic conditions

Chromatographic separations of liver samples were performed on Waters ACQUITY UPLC HSS T3 C18 RRHD column (100 nm × 2.1 mm, 1.8 μm) (Waters Corp, Milford, MA, USA) at 55°C; The mobile phase A: acetonitrile-water (60:40, V/V), containing 0.1%FA and 10 mM NH_4_COOH; The mobile phase B: acetonitrile-isopropanol (10:90, V/V), containing 0.1% FA and 10 mM NH_4_COOH; The flow rate: 0.35 mL/min; Sample volume: 5 μL. The elution gradient is shown in Supplementary Table 1.

##### Mass spectrometry conditions

The mass spectrometry system adopts Qtrap6500 plus mass spectrometry system of American AB Sciex company, which is equipped with electrospray ionization (ESI) ion source and Analyst1.7 workstation. The optimized mass spectrometry conditions are as follows: air curtain gas, 40 psi; Ion spray voltage, −4,500/5,500 v; Source temperature, 400°C; Atomizing gas, 50 psi; Auxiliary heating gas, 55 psi. Schedule-MRM mode was used for high-throughput analysis of more than 1,000 lipids.

#### Data analysis

Qualitative analysis of MRMPROBS: The liver samples were detected by a high-performance liquid chromatography (AB Exion LC) and a highly sensitive mass spectrometer (Qtrap 6500 plus) to obtain original off-board data. The automatic batch processing such as peak extraction, peak alignment, peak identification, and peak area integration was performed by the software MRMPROBS. The relevant parameter settings are as follows: Smoothing level, 2; Minimum peak width, 5; Minimum peak height, 500; Retention time tolerance, 0.2 min. Finally, the derived qualitative and quantitative tables were analyzed quantitatively by response factor method.

##### Qualitative and quantitative results

After the integral peak area of metabolites is brought into the calculation formula, the semi-quantitative data of each metabolite in the actual sample is finally obtained. The quantitative formula is as follows:

The lipid content (ng/mL or g) = A1/A2*C*V/N

A1: peak area of target lipid; A2: the peak area of lipid internal standard corresponding to the target lipid; C: adding the internal standard concentration value (ng/mL) corresponding to the lipid internal standard into the sample; V: constant volume (0.2 mL); N: weigh the sample quality.

##### Multivariate statistical analysis

Unsupervised principal component analysis (PCA) was used to observe the overall distribution among samples and the stability of the whole analysis process, and then supervised partial least squares (PLS-DA) and orthogonal partial least squares (OPLS-DA) were used to distinguish the overall differences of metabolic profiles among groups and identify the differential metabolites. According to OPLS-DA model, the variable weight value (VIP) was obtained, where VIP > 1 found the potential biomarker. The larger VIP indicated that the variable had a greater contribution to the subgroup.

##### Univariate statistical analyses

Univariate analysis focuses on the description and statistical inference of univariate data and describes the concentrated or discrete trends in sample data. Univariate statistical inference is to infer the overall situation from the sample data, including interval estimation and statistical hypothesis testing. Student’s test and Fold change analysis are often used to compare the metabolites between two groups.

#### Screening of differential metabolites

A combination of multi-dimensional and single-dimensional analyses were performed to screen the differential metabolites between groups. In OPLS-DA analysis, VIP value could be used to measure the strength and explanation ability of the expression pattern of each metabolite on the classification and discrimination of each group of samples, and to dig for biologically significant differential metabolites. The *t*-test was then used to verify whether the differential metabolites between the groups were significant. The screening criteria were as followed: VIP value of first principal component of OPLS-DA model was greater than 1, and *p*-value of *T*-test was less than 0.05. Metabolic pathway enrichment analysis of differential metabolites was performed based on the KEGG database.

### Cecal contents metagenomic sequencing

#### DNA extraction and library preparation

With a MagPure Soil DNA KF kit (MGBio), total DNA has been extracted from cecal contents. After the sample DNA was qualified in the detection, DNA was fragmented by ultrasound. Then, the fragmented DNA was purified, the ends were repaired and the sequencing adapter was ligated. DNA fragments were adenylate 3′ ends after end repair. Fragment size selection was performed by agarose gel electrophoresis and PCR amplification was performed to form a sequencing library. First, the quality of the constructed library was checked, and the qualified library was sequenced on the Illumina sequencing platform. Library construction and metagenome sequencing were conducted by OE biotech Co., Ltd. (Shanghai, China).

#### Bioinformatics

The raw data was in FASTQ format. Reads were trimmed and filtered using Trimmomatic (v0.36)1. Host pollution control was needed if the DNA was extracted from host-related environment. The post-filtered pair-end reads were aligned against the host genome using bowtie2 (v2.2.9)2 and the aligned reads were discarded. Metagenome assembly was performed using MEGAHIT (v1.1.2)3 after getting valid reads. Use gaps inside scaffold as breakpoint to interrupt the scaffold into new contigs (Scaftig), and these new Scaftig with length ≧ 500 bp of were retained. ORF prediction of assembled scaffolds using prodigal (v2.6.3)4 was performed and translated into amino acid sequences. The non-redundant gene sets were built for all predicted genes using CDHIT (v4.6.7)5. The clustering parameters were 95% identity and 90% coverage. The longest gene was selected as representative sequence of each gene set. Clean reads of each sample were aligned against the non-redundant gene set (95% identity) using bowtie2 (v2.2.9), and the abundant information of the gene in the corresponding sample was counted. The gene set representative sequence (amino acid sequence) was annotated with NR, KEGG, COG, SWISSPROT, GO database with an *e*-value of 1e-5. The taxonomy of the species was obtained as a result of the corresponding taxonomy database of the NR Library, and the abundance of the species was calculated using the corresponding abundance of the genes. In order to construct the abundance profile on the corresponding taxonomy level, abundance statistics were performed at each level of Phylum, Order, and Species. The gene sets were compared with the CAZy database using the corresponding tool hmmscan (v3.1b2) to obtain the information of the carbohydrate active enzyme corresponding to the gene and then calculated the carbohydrate activity using the sum of the gene abundances corresponding to the carbohydrate active enzyme abundance. The PCA analysis and plotting of the abundance spectrum of the species abundance spectrum were carried out using R software (v3.2.0), and the results of the equidistant matrix of PCoA and NMDS were calculated and analyzed.

### Statistical analysis

The statistical analysis was performed using SPSS software (version 19.0 SPSS). The differences among groups were analyzed by one-way ANOVA followed by a Dunnett *post hoc* test and then Student’s test was used to comparison between two groups. *P*-values below 5% were considered significant. The experimental data were expressed as means ± SD.

## Results

### Effect of aspirin eugenol ester on mice blood lipids and body weight

The results of blood lipids were shown in Table 1. Levels of TCH and LDL-C were higher in the ApoE−/− + HFD group than those in the C57 + ND group (*P* < 0.01). In comparison with the ApoE−/− + HFD group, TCH level in the AEE H group was decreased (*P* < 0.05) and was significant decreased in the AEE M group and in the AEE L group (*P* < 0.01). Compared with ApoE−/− + HFD group, LDL-C level of AEE H, AEE M, and AEE L groups were all significantly decreased (*P* < 0.01). The result of body weight of mice in each group were shown in Supplementary Figure 1.

**TABLE 1:** Effect of AEE on blood lipids in mice.

Variables (mmol/L)	C57 + ND	ApoE−/− + HFD	AEE H	AEE M	AEE L
TCH	3.78 ± 0.43	32.61 + 2.69**	29.59 ± 3.16^#^	27.50 + 2.10^##^	26.22 ± 2.58^##^
LDL-C	0.21 ± 0.01	7.56 + 1.24**	5.51 ± 1.11^##^	5.10 + 1.16^##^	5.28 ± 1.00^##^

ND, Normal diet; HFD, high-fat diet; AEE, aspirin eugenol ester; C57 + ND, C57BL/6J + ND; AEE H, ApoE−/− + HFD + AEE (H: 217 mg/kg/d); AEE M, ApoE−/− + HFD + AEE (M: 168 mg/kg/d); AEE L, ApoE−/− + HFD + AEE (L: 118 mg/kg/d); TCH, total cholesterol; LDL-C, low density lipoprotein-cholesterol. Data are expressed as the means ± SD (*n* = 6).

**P < 0.01 compared with the C57 + ND group; ^##^*P* < 0.01 compared with the ApoE−/− + HFD group; ^#^*P* < 0.05 compared with the ApoE−/− + HFD group.

### Total ion chromatogram of liver lipidomics

TIC is a plot of the time or number of scans taken as a synthesis of the intensities of all ions over a selected mass. The result was shown in Supplementary Figure 2. Under the optimized chromatographic and mass spectrometric conditions, the peak line of TIC is sharp. Metabolites in liver samples were effectively separated with high detection degree for metabolites in samples.

### The number of lipid species in lipid classes

**FIGURE 1:**
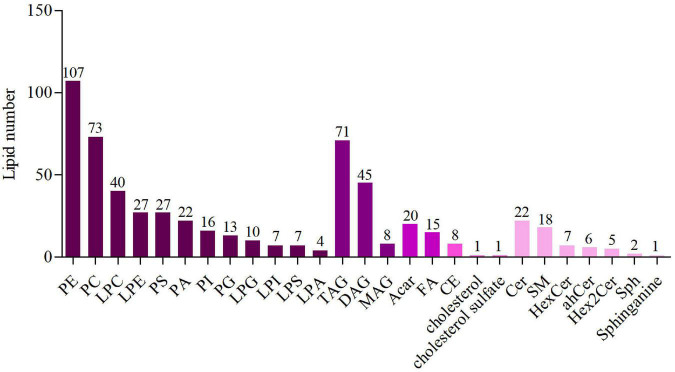
The number of lipid species in lipid classes. Different colors represent different lipid classes and the numbers represent the number of lipid species.

In this study, lipid compounds were divided into 5 types, including Glycerophospholipids, Glycerolipids, Fatty acyls, Sterol lipids, Sphingolipids. In each class type, there were different subtypes with polarity at the head (lipid class). For each subgroup, different molecular species (lipid species) of lipid compounds were classified based on differences that couldn’t be explained by saturation or length of carbon chains. Overall, lipid compounds were classified into three levels. The results were shown in Figure 1. The lipidomic analysis of mice liver revealed 583 lipid species sorted into 26 lipid classes, and the specific classification was as followed: Glycerophospholipids: phosphatidylethanolamine (PE), phosphatidylcholine (PC), lysophosphatidylcholine (LPC), Lysophosphatidyl ethanolamine (LPE), phosphatidylserine (PS), phosphatidic acid (PA), phosphatidylinositol (PI), phosphatidylglycerol (PG), Lysophosphatidylglycerol (LPG), Lysophosphatidyl inositol (LPI), Lysophosphatidylserine (LPS), lysophosphatidic acid (LPA); Glycerolipids: triacylglycerol (TAG), diacylglycerol (DAG), monoglyceride (MAG); Fatty acyls: acyl-carnitine (Acar), fatty acids (FA); Sterol lipids: cholesterol ester (CE), cholesterol sulfate; Sphingolipids: ceramide (Cer), sphingomyelin (SM), Hexoside ceramide (HexCer), Dihydroceramide (dhCer), Hex2Cer, Sphingosine (Sph), Sphinganine. In this study, the number of lipid species as glycerophospholipids was the most, followed by glycerolipids.

### Aspirin eugenol ester improved lipid metabolism in the liver by lipidomics analysis

**FIGURE 2:**
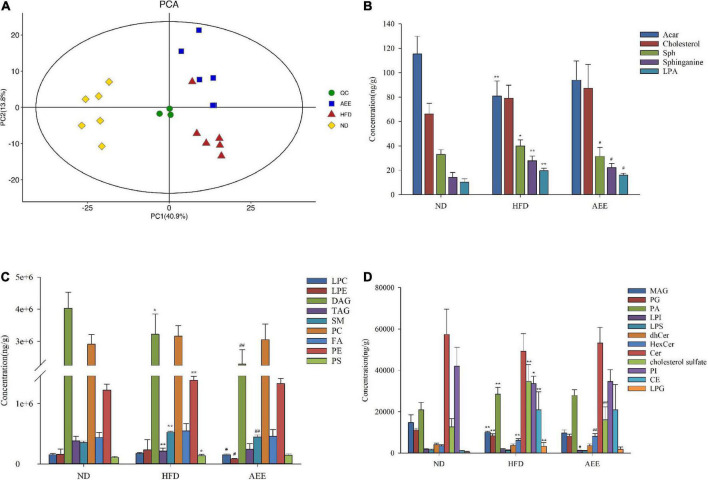
Lipidomics analysis of mice liver samples. **(A)** PCA score plots of liver lipid profiling on all samples. **(B–D)** The concentration of different lipid compositions in the liver. Data are expressed as the means ± SD (*n* = 6). Differences were assessed by ANOVA and denoted as follows: **P < 0.01, *P < 0.05 compared with the ND group; ^##^*P* < 0.01, ^#^*P* < 0.05 compared with the HFD group.

In order to determine the lipid composition of the liver in these groups, we performed liver lipidomics. By using lipidomics, 583 species as well as 26 classes of lipids were analyzed, including 40 LPCs, 27 LPEs, 20 PAs, 107 PEs, 45 DAGs, 71 TAGs, 74 PCs, 22 Cers, 18 SM, 12 HexCer, and others (Supplementary File 1). As shown in Figures 2B–D, in comparison with the ND group, LPAs, cholesterol sulfate, SMs, and Sphinganing concentrations in HFD group were significantly increased (*P* < 0.01). However, compared with HFD group, these lipid concentrations were decreased in the AEE group. Comparatived to the ND group, PGs, PIs, MAGs, TAGs, and ACars concentrations decreased, while LPGs, PAs, PEs, and CEs concentrations increased. The results showed that AEE could improve the lipid metabolism disorders in mouse liver induced by high-fat diet. PCA was performed on all samples, and the QC samples were closely clustered in the center of the PCA score plot, suggesting high repeatability and stable performance throughout the LC-MS analysis as depicted in Figure 2A.

### Orthogonal partial least squares-DA and volcano plot analysis of lipid in the liver

**FIGURE 3:**
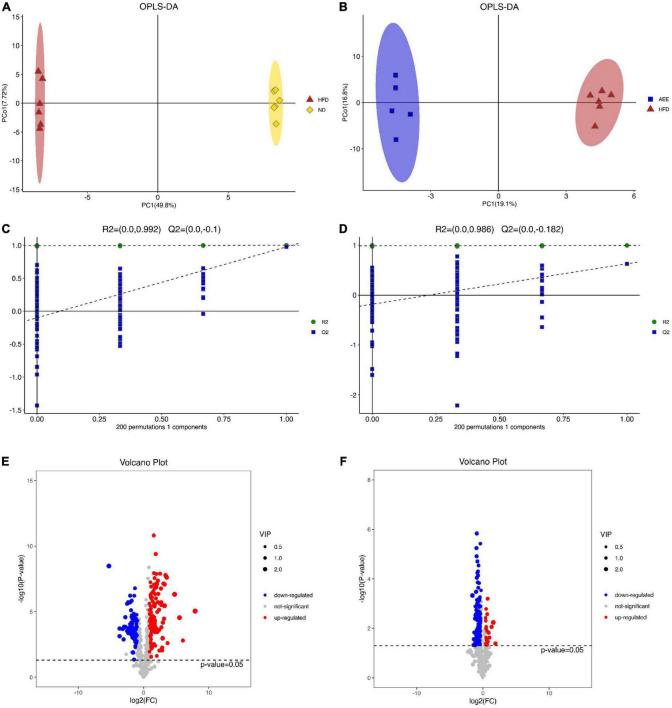
OPLS-DA and volcano plot analysis of lipid in the liver. **(A)** OPLS-DA analysis in HFD vs. ND. **(B)** OPLS-DA analysis in AEE vs. HFD. **(C)** OPLS-DA displacement test of HFD vs. ND (Q2 = –0.1). **(D)** OPLS-DA displacement test of AEE vs. HFD (Q2 = –0.182). **(E)** Volcano plot of differentially lipids in HFD vs. ND. **(F)** Volcano plot of differentially lipids in AEE vs. HFD. Red dots represent significantly upregulated lipids and blue dots represent significantly downregulated lipids. Lipids that are not significant are represented by gray dots.

As shown in Supplementary Figures 3A,B, the separation between the ND group and HFD group was clearly, while samples from HFD and AEE groups partially overlapped in PCA. In order to achieve further separation of groups and increase discrimination between them, PLS-DA (Supplementary Figures 3C,D) and OPLS-DA (Figure 3A,B) models were also applied.

The samples in the ND group and the HFD group were completely separated and far away, indicating that the samples in the two groups had little similarity, further indicating that the high-fat diet and ApoE gene knockout caused great changes in the lipids of the mouse liver. Similarly, the samples from the HFD and AEE groups were completely separated and far apart, indicating that there was little similarity between the two groups of samples, further reflecting the large changes in the hyperlipidemia state of mice after AEE treatment. Moreover, based on the reduction of serum LDL-C and TCH levels after AEE intervention, it indicated that AEE intervention relieved the hyperlipidemia in mice. As shown in Figures 3C,D, the value of Q2 is less than 0 and the OPLS-DA displacement test demonstrated that these OPLS-DA were not overfitting of test model.

In order to visualize the overall distribution of differentially lipids, a volcano plot was used. Different lipids were assigned based on VIP > 1 and adjusted *P* < 0.05. Positive Log2FC values indicated lipids up-regulation, and negative Log2FC values indicated lipids down-regulation. As shown in Figures 3E,F, there were 198 different lipids in the HFD group compared with ND group. The AEE group discovered 139 different lipids compared with the HFD group.

### Aspirin eugenol ester altered the important differential lipid species in the liver

**FIGURE 4:**
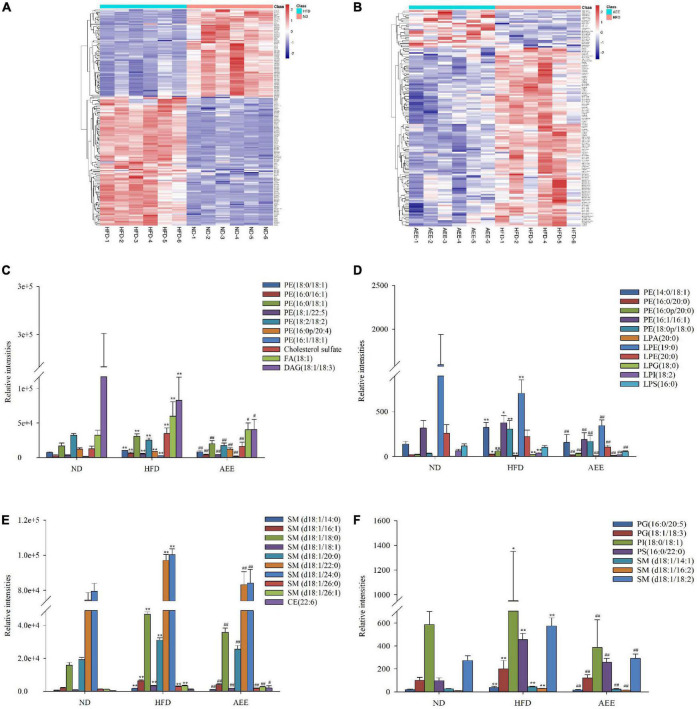
AEE altered the important differential lipid species in the liver. **(A)** Different lipids in HFD vs. ND. **(B)** Different lipids in AEE vs. HFD. **(C–F)** The important hepatic differential lipid species in the HFD and AEE groups. Data are expressed as the means ± SD (*n* = 6). Differences were assessed by ANOVA and denoted as follows: **P < 0.01, *P < 0.05 compared with the ND group; ^##^*P* < 0.01, ^#^*P* < 0.05 compared with the HFD group.

On the heatmap of Figure 4A, there were 198 different lipids in the HFD group compared with ND group and the upregulated lipids were focused mainly on LPCs, PEs, PCs, SMs, and LPGs, but downregulated lipids were focused mainly on TAGs, Cers, DAGs, PEs, and PGs. There were 139 different lipids in the AEE group compared with HFD group and the upregulated lipids were concentrated mainly on TAGs and PEs, but downregulated lipids were concentrated mainly on LPCs, Cers, SMs, DAGs, LPEs, and PEs in Figure 4B. These results suggest that AEE intervention alleviate the disorder of liver lipid metabolism principally by regulation of LPCs, PEs, SMs, TAGs, and so on. To identify specific lipid biomarkers involved in HFD and AEE, we analyzed the major and remarkable differential lipid species.

As shown in Figures 4C–F, the concentrations of LPA (20:0), LPG (18:0), 10 PEs [PE (14:0/18:1), PE (16:0/16:1), PE (16:0/18:1), PE (16:0/20:0), PE (16:0p/20:0), PE (16:1/16:1), PE (16:1/18:1), PE (18:0/18:1), PE (18:0p/18:0), and PE (18:0/22:5)], two PGs [PG (16:0/20:5) and PG (18:1/18:3)], PI (18:0/18:1), PS (16:0/22:0), 12 SMs [SM (d18:1/14:0), SM (d18:1/14:1), SM (d18:1/16:1), SM (d18:1/16:2), SM (d18:1/18:0), SM (d18:1/18:1), SM (d18:1/18:2), SM (d18:1/20:0), SM (d18:1/22:0), SM (d18:1/24:0), SM (d18:1/26:0), and SM (d18:1/26:1)], Cholesterol sulfate, FA (d18:1) in the HFD group were significantly increased (*P* < 0.01) compared to the ND group, while AEE treatment observably decreased the concentration of these lipids. Hence, these lipids were likely to be potential biomarkers for AEE to regulate lipid metabolism in hyperlipidemia mice. However, in contrast to the ND group, the concentrations of [LPE (19:0), LPI (18:2), PE (18:2/18:2), and DAG (18:1/18:3)] observably decreased in the HFD group (*P* < 0.01) and AEE treatment degraded these lipids (*P* < 0.01).

### Pathway analysis of liver lipidomics

**FIGURE 5:**
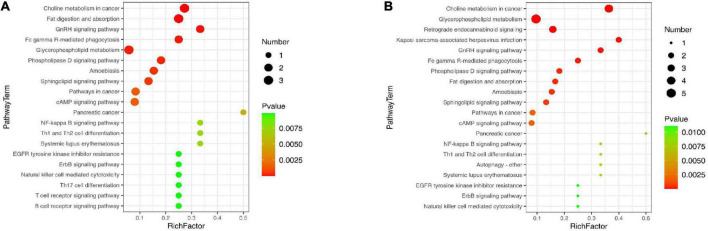
Lipid metabolism pathway analysis based on KEGG enrichment map for significantly different lipid species in HFD vs. ND **(A)** and AEE vs. HFD **(B)**. In the metabolic pathway *P*-value was the significance of the metabolic pathway, and the significance-enriched pathway was selected for bubble mapping. The ordinate was the name of the metabolic pathway and the abscissa was the Rich factor. The larger the Rich Factor was, the greater the enrichment would be. The color from green to red indicated that *P*-value decreased in turn, and the lower *P*-value represents the more significant degree of enrichment. A larger dot indicates a greater number of metabolites were enriched on this pathway.

In order to further explore the effects of AEE on the metabolic pathways involved in differential lipid species in the liver with the process of alleviating hyperlipidemia in mice, enrichment analysis of metabolic pathways of differential lipid species was performed based on KEGG database. As shown in Figure 5A, compared with the ND group, the pathways of choline metabolism in cancer, fat digestion and absorption, GnRH signaling pathway, glycerophospholipid metabolism, sphingolipid signaling pathway, cAMP signaling pathway, NF-kB signaling pathway, Th1 and Th2 cell differentiation, T/B cell receptor signaling pathway were destroyed in the model group. However, AEE treatment ameliorated the pathways of choline metabolism in cancer, glycerophospholipid metabolism, GnRH signaling pathway, fat digestion and absorption, sphingolipid signaling pathway, cAMP signaling pathway, NF-kB signaling pathway, Th1 and Th2 cell differentiation in Figure 5B.

### Gene level analysis of metagenomic sequence

Trimmomatic was used to preprocess the raw data obtained by sequencing Illumina, and the number of reads was counted. Subsequently, MEGAHIT was used for sequence assembly to obtain contigs, and more than 500 bp contigs were screened for statistics. The ORF prediction of spliced contigs sequences was performed using Prodigal, followed by redundancy removal using CD-HIT to obtain the unigene.

According to the number of reads and gene length on the comparison, the abundance information of each gene in each sample was calculated, and the calculation formula was shown in Supplementary Figure 4B. The results of gene abundance calculation are shown in Supplementary File 2. The Core and Pan genes dilution curves in Supplementary Figure 4A demonstrated that the amount of data was reliable and stable. Venn graph of the number of genes for all samples was shown in Supplementary Figure 4C. The total number of genes shared by all samples was 256,650. There was a large difference in the number of genes between the HFD group and AEE. In order to investigate the gene number between groups, a violin diagram of gene number was drawn, and the results were shown in Supplementary Figure 4D. There were significant differences in the number of genes between the HFD group and AEE group (*P* < 0.01).

### Species level analysis of metagenomic sequence

DIAMOND was used to compare unigene with the NR pool of NCBI and the abundance of the species was calculated using the sum of the gene abundance of the corresponding species. The abundance of species in each sample was counted at the Phylum, Order, and Species levels to construct abundance profile at the corresponding taxonomic levels.

**FIGURE 6:**
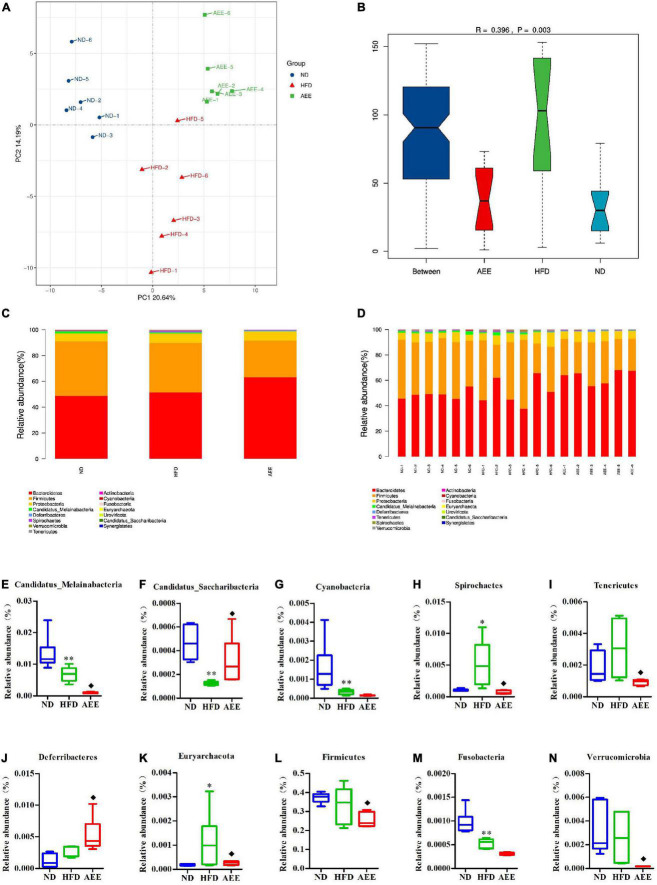
Microbiota compositions at the Phylum level. **(A)** PCA results of the similarities in bacterial community structures at the Phylum level. **(B)** Analysis of Anosim based on Phylum level. **(C,D)** Histogram of relative abundance at the Phylum level. **(E–N)** Relative content of Candidatus_Melainabacteria, Candidatus_Saccharibacteria, Cyanobacteria, Deferribacteres, Euryarchaeota, Firmicutes, Fusobacteria, Spirochetes, Tenericutes, and Verrucomicrobia at the Phylum level. Values are presented as mean ± SD (*n* = 6). Differences were assessed by ANOVA and denoted as follows: ^**^P < 0.01, *P < 0.05 compared with the ND group; ^◆^*P* < 0.05 compared with the HFD group.

**FIGURE 7:**
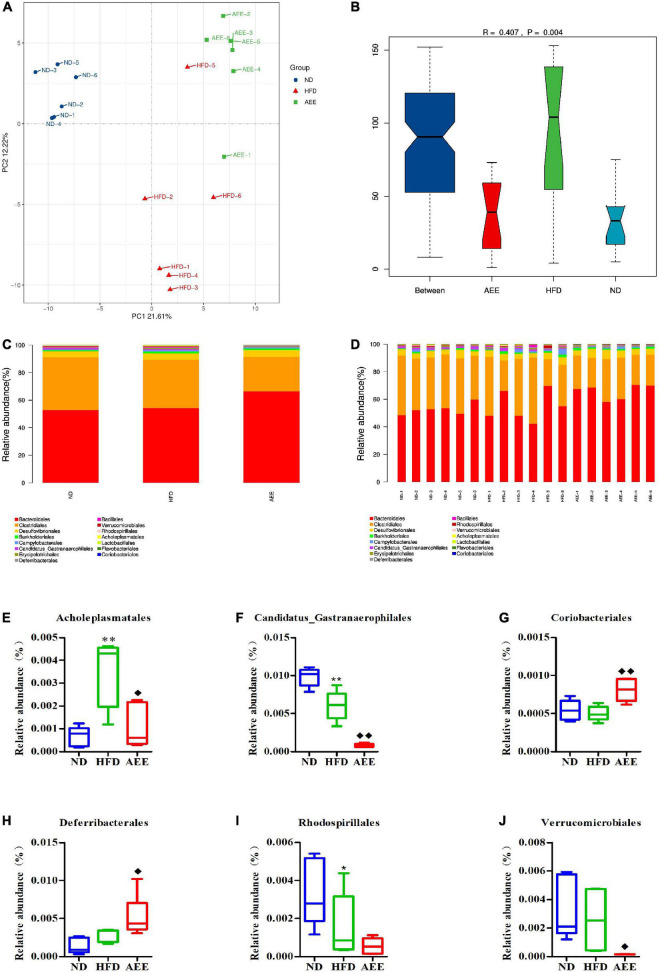
Microbiota compositions at the Order level. **(A)** PCA results of the similarities in bacterial community structures at the Order levels. **(B)** Analysis of Anosim based on Order level. **(C,D)** Histogram of relative abundance at the Order level. **(E–J)** Relative content of Acholeplasmatales, Candidatus_Gastranaerophilales, Coriobacteriales, Deferribacterales, Rhodospirillales, and Verrucomicrobiales at the Order level. Values are presented as mean ± SD (*n* = 6). Differences were assessed by ANOVA and denoted as follows: ^**^P < 0.01, *P < 0.05 compared with the ND group; ^◆ ◆^*P* < 0.01, ^◆^*P* < 0.05 compared with the HFD group.

**FIGURE 8:**
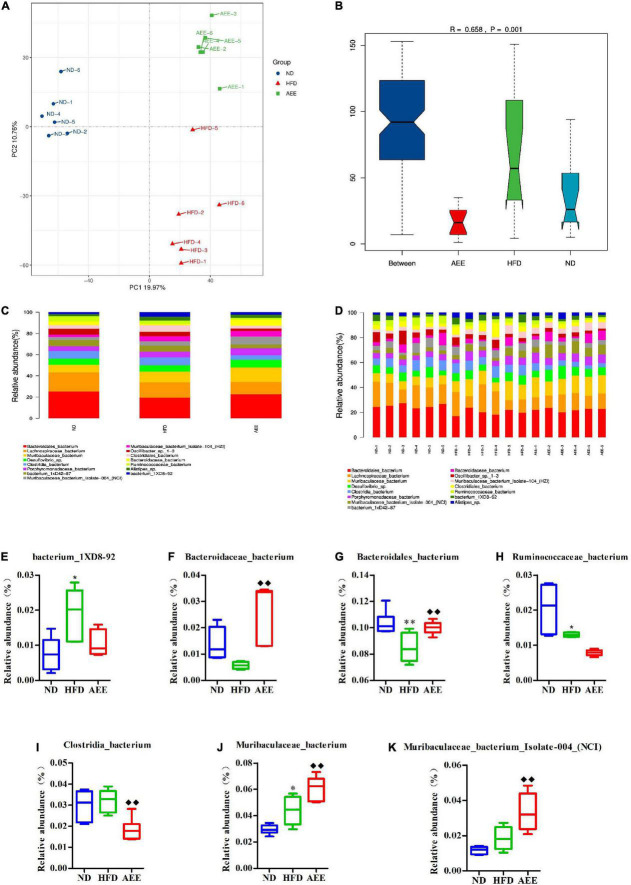
Microbiota compositions at the Species level. **(A)** PCA results of the similarities in bacterial community structures at the Species level. **(B)** Analysis of Anosim based on Species level. **(C,D)** Histogram of relative abundance at the Species level. **(E–K)** Relative content of bacterium_1XD8-92, Bacteroidaceae_bacterium, Bacteroidales_bacterium, Clostridia_bacterium, Muribaculaceae_bacterium, Muribaculaceae_bacterium_Isolate-004_(NCI), and Ruminococcaceae_bacterium at the Species level. Values are presented as mean ± SD (*n* = 6). Differences were assessed by ANOVA and denoted as follows: ^**^P < 0.01, *P < 0.05 compared with the ND group; ^◆ ◆^*P* < 0.01 compared with the HFD group.

As shown in Figures 6–8A, the PCA was used to analyze the similarities in bacterial community structures among ND, HFD, and AEE groups of samples at the Phylum, Order, and Species levels. Significant changes were noted in both the HFD and AEE intervention groups. At the three levels, the samples of the ND group were far from the HFD group samples, and there were significant differences in bacterial community structures. Likewise, there are obvious differences in the bacterial community between the samples of HFD and AEE groups. Results demonstrated that high-fat diet induced significant changes in bacterial community structures of mice, and AEE treatment partially restored the bacterial community.

As shown in Figures 6–8B, the Anosim analysis was used to test whether differences in bacterial communities between groups were significantly greater than within groups at the Phylum, Order, and Species levels. *R*-values higher than 0 indicate that the difference between groups is greater than the difference within the group. The difference within group with R less than 0 was greater than that between group. *P* < 0.05 indicated the statistical significance. At the Phylum, Order, and Species levels, the inter-group differences in bacterial communities were extremely significant (*R* = 0.396, *P* = 0.003; *R* = 0.407, *P* = 0.004; *R* = 0.658, *P* = 0.001).

The histograms show species with TOP15 abundance by Phylum, Order, and Species levels in Figures 6–8C,D. At the Phylum level in Figures 6C,D, Bacteroidetes has the largest abundance fraction, followed by Firmicutes, Proteobacteria, and so on.

Compared to ND group, the abundances of Candidatus_Melainabacteria, Candidatus_Saccharibacteria, Cyanobacteria, and Fusobacteria were significantly decreased (*P* < 0.01) and the abundance of Spirochetes was significantly increased (*P* < 0.01) in the HFD group. AEE treatment increased the abundance of Deferribacteres and Candidatus_Saccharibacteria (*P* < 0.05) and decreased the abundances of Firmicutes, Candidatus_Melainabacteria, Tenericutes, Spirochetes, Verrucomicrobia, and Euryarchaeota (*P* < 0.05) in comparison with the HFD group in Figures 6E–N. At the Phylum level, Bacteroidetes has the largest abundance fraction, followed by Firmicutes, and AEE decreased the abundance of Firmicutes, but had no effect on the abundance of Bacteroidetes.

At the Order level in Figures 7C,D, Bacteroidales has the highest abundance fraction, followed by Clostridiales and Desulfovibrionales. In comparison with the ND group, the abundance of Candidatus_Gastranaerophilales significantly decreased (*P* < 0.01), Rhodospirillales reduced (*P* < 0.05) and Acholeplasmatales increased considerably in the HFD group (*P* < 0.01). AEE treatment greatly decreased the abundance of Candidatus_Gastranaerophilales and increased the abundance of Coriobacteriales (*P* < 0.01). Likewise, AEE treatment elevated the abundance of Deferribacterales and decreased the abundances of Verrucomicrobiales and Acholeplasmatales (*P* < 0.05) in Figures 7E–J.

At the species level in Figures 8C,D, Bacteroidales_bacterium has the highest abundance fraction, followed by Lachnospiraceae_bacterium and Muribaculaceae_bacterium. Compared to ND group, the abundance of Bacteroidales_bacterium significantly decreased (*P* < 0.01), Ruminococcaceae_bacterium reduced (*P* < 0.05), Muribaculaceae_bacterium, and bacterium_1XD8-92 increased (*P* < 0.05) in the HFD group. AEE treatment significantly elevated (*P* < 0.01) the abundance of Bacteroidales_bacterium, Muribaculaceae_bacterium, Muribaculaceae_bacterium_Isolate-004_(NCI) and Bacteroidaceae_bacterium, and dramatically decreased (*P* < 0.01) the abundance of Clostridia_bacterium in comparison with HFD group in Figures 8E–K.

**FIGURE 9:**
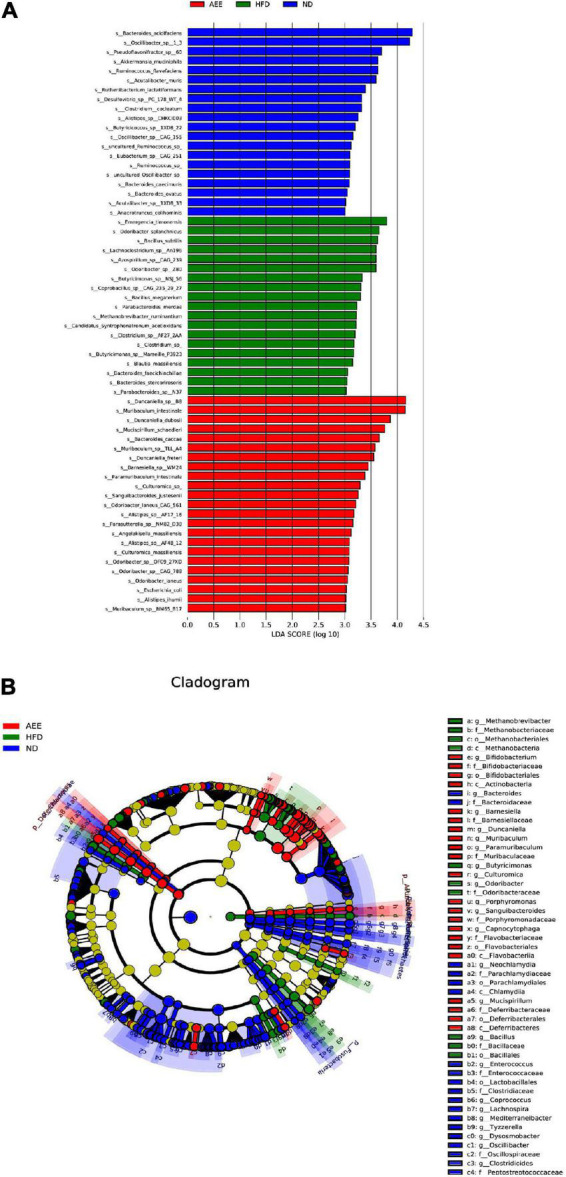
The analysis of linear discriminant analysis coupled with effect size measurements (LEfSe). **(A)** The linear discriminant analysis (LDA). Different colors indicated different groups, the blue, green, and red bars indicated species with relatively high abundance in the ND, HFD, and AEE groups, respectively (LDA). **(B)** Differential species annotation branch diagram. Differential species annotation branch illustration. Different colors indicated different subgroups. The blue, green, and red nodes indicated differentially significant species with relatively high abundance in the ND, HFD, and AEE groups, and the yellow nodes indicated species with no significant difference in the comparison between the two groups (HFD vs. ND; AEE vs. HFD). The node diameter was in direct proportion to the relative abundance. The nodes in each layer represented Phylum, Order, and Species, respectively, from inside to outside. The notes for species markers in each layer represented Phylum, Order, and Species from outside. The lettered species names are shown in the legend on the right.

As showcased in Figure 9, the linear discriminant analysis (LDA) coupled with effect size measurements (LEfSe) was used to identify the difference in bacterial community composition as biomarkers between the ND and HFD group, the HFD and the AEE group. Different colors of blue, green and red represent the ND, HFD, and AEE groups, respectively. LDA more than 2 reflects significant difference between groups. A higher LDA value represents a greater contribution to the difference between groups, which indicates that this microflora is a more important biomarker. As shown in Figure 9A, Ruminococcaceae, Oscillospiraceae, and Oscillibacter were species with relatively high abundance in the ND group. Odoribacteraceae, Alphaproteobacteria, and Rhodospirillales were species with relatively high abundance in the HFD group. Muribaculaceae, Duncaniella, and Muribaculum were species with relatively high abundance in the AEE group. To find out the biomarker microbiota with statistical abundance differences between the different groups, LEfSe analysis was performed. As shown in Figure 9B, these species at six taxonomic levels (from Phylum to Species), P__Deferribacteres, C__Deferribacteres, O__Deferribacterales, F__Deferribacteraceae, G__Mucispirillum, and S__*Mucispirillum_schaedleri*, were markedly different species in AEE group (*P* < 0.01). These species at five taxonomic levels (from Class to Species), C__Flavobacteriia, O__Flavobacteriales, F__Flavobacteriaceae, G__Capnocytophaga, and S__*Capnocytophaga_felis*, were different species in AEE group (*P* < 0.05). These species at six taxonomic levels (from Phylum to Species), P__Actinobacteria, C__Actinobacteria, O__Bifidobacteriales, F__Bifidobacteriaceae, G__Bifidobacterium, and S__*Bifidobacterium_adolescentis* were different species in AEE group (*P* < 0.05). Notably, almost all species with significant changes in the AEE group were members of the Bacteroidetes (78%) and a small portion of the Firmicutes (12%). Thus, these results suggested that AEE mainly improved the hyperlipidemia of mice by changing the flora of Deferribacteres, Actinobacteria, Flavobacteriia, Bacteroidetes, and Firmicutes.

### Functional level analysis of metagenomic sequence

There are five functional annotation systems used: eggNOG, KEGG, CAZy, CARD, GO in Supplementary Figure 5. Functional annotation results of eggNOG showed that some genes were involved in lipid transport and metabolism. Based on KEGG-functional annotation, some genes were enriched in cardiovascular disease, immune disease, lipid metabolism, energy metabolism, and so on. The annotation results of the CAZy database showed that glycoside hydrolase, glycosyltransferase, and carbohydrate esterases were the first three carbohydrate enzymes. The annotation results of the CARD database showed that the relative content of resistance genes corresponding to peptide and rifamycin of antibiotic resistance was high, followed by lincosamide and glycopeptide. GO annotated three categories, including biological process, cellular component and molecular function.

**FIGURE 10:**
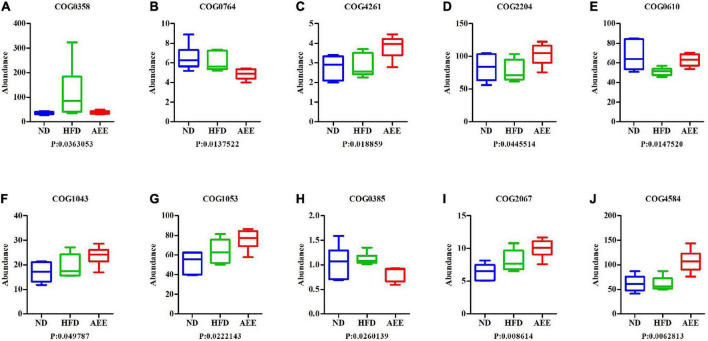
The significant difference functions in eggNOG. **(A–J)** The significant difference functions in eggNOG are DNA primase activity, unsaturated fatty acids biosynthesis, lipid A biosynthesis acyltransferase, two component, sigma 54 specific, transcriptional regulator, fis family, type site-specific deoxyribonuclease, the biosynthesis of lipid A, succinate dehydrogenase, bile acid, lipid transport and transposase. The horizontal axis is the sample grouping; Vertical is the relative abundance of the corresponding function. *P* < 0.05 indicated significant difference between the two groups, and *P* < 0.01 indicated extremely significant difference between the two groups.

**FIGURE 11:**
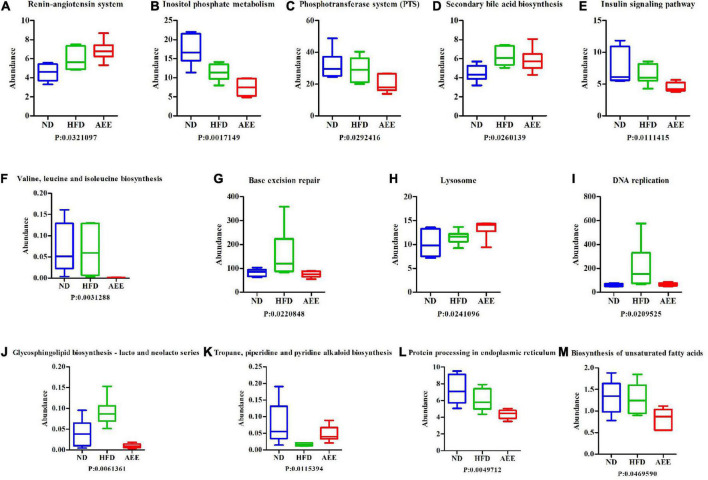
The significant difference functions in KEGG. **(A–M)** The significant difference functions in KEGG are renin-angiotensin system, inositol phosphate metabolism, phosphotransferase system, secondary bile acid biosynthesis, insulin signaling pathway, Valine, leucine and isoleucine biosynthesis, base excision repair, lysosome, DNA replication, glycosphingolipid biosynthesis, tropane, piperidine and pyridine alkaloid biosynthesis, protein processing in endoplasmic reticulum, and biosynthesis of unsaturated fatty acids. The horizontal axis is the sample grouping; Vertical is the relative abundance of the corresponding function. *P* < 0.05 indicated significant difference between the two groups, and *P* < 0.01 indicated extremely significant difference between the two groups.

In order to investigate whether there were significant differences in function among different groups, Kruskal-Wallis method was used to test the hypothesis of function abundance data among groups to obtain *P*-value, where *P* was less than 0.05 and it was considered to have significant difference. We screened the abundance information of the functions with significant difference based on the *P*-value. In the eggNOG, the significant difference function boxplot was shown in Figure 10. The significant difference functions in that eggNOG were shown in Table 2. As shown in Figures 10A–J, these results showed that the significant difference functions were mainly lipid transport, the biosynthesis of lipid A, bile acid, unsaturated fatty acids biosynthesis, and DNA primase activity, etc. In the KEGG, the significant difference function boxplots were shown in Figure 11. Results demonstrated that the significant difference functions were mainly base excision repair, biosynthesis of unsaturated fatty acids, glycosphingolipid biosynthesis, insulin signaling pathway, inositol phosphate metabolism, secondary bile acid biosynthesis, Valine, leucine and isoleucine biosynthesis, and so on in Figures 11A–M.

**TABLE 2:** Difference function in EggNOG.

EggNOG_ID	EggNOG_description
COG2067	Lipid transport
COG1043	The biosynthesis of lipid A
COG4261	Lipid A biosynthesis acyltransferase
COG0385	Bile acid
COG0764	Unsaturated fatty acids biosynthesis
COG1053	Succinate DeHydrogenase
COG2204	Two component, sigma54 specific, transcriptional regulator, Fis family
COG4584	Transposase
COG0610	Type I site-specific deoxyribonuclease
COG0358	DNA primase activity

### Association analysis of cecal metagenomics and liver lipidomics

**FIGURE 12:**
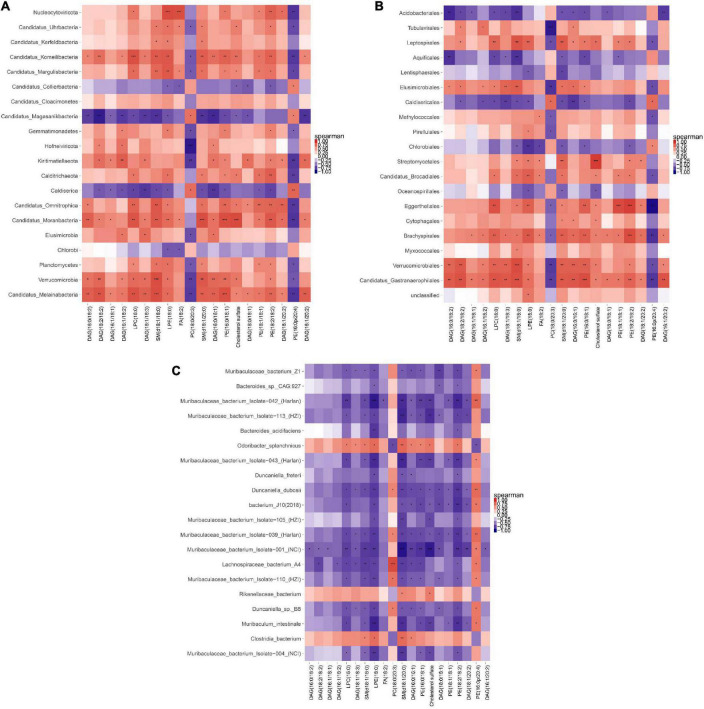
Correlation heat map of metagenomic and lipidomics. **(A)** Correlation analysis at the Phylum level. **(B)** Correlation analysis at the Order level. **(C)** Correlation analysis at the species level. For each species/gene that behaves differently, each column is listed as the corresponding metabolite. The orange color showed a positive correlation, while the blue color showed a negative correlation. The deeper the color was, the greater the correlation would be. The closer the color was to white, the closer the correlation would be to zero. ***P < 0.001; **P < 0.01; *P < 0.05.

A correlation heat map shown in Figure 12 was drawn based on the Top20 results from the association analysis between differential species/gene and differential metabolite. Lipidomics results showed that AEE treatment observably decreased the concentration of PE (16:0/18:1), SM (d18:1/18:0), SM (d18:1/20:0) and Cholesterol sulfate (*P* < 0.01), and increased the concentration of DAG (18:1/18:3) compared to the HFD group. At the Phylum level in Figure 12A, AEE treatment decreased (*P* < 0.05) the abundance of Candidatus_Melainabacteria and Verrucomicrobia in comparison with the HFD group. The results of the joint analysis showed that compared with the HFD group, in the AEE group the abundance of Verrucomicrobia was significantly positively correlated with the concentration of SM (d18:1/18:0) (*P* < 0.001), positively correlated with the concentrations of SM (d18:1/20:0) and PE (16:0/18:1) (*P* < 0.01), positively correlated with the concentrations of DAG (18:1/18:3) and Cholesterol sulfate (*P* < 0.05). Likewise, in the AEE group the abundance of Candidatus_Melainabacteria was significantly positively correlated with the concentrations of SM (d18:1/18:0) and PE (16:0/18:1) (*P* < 0.001), positively correlated with the concentrations of DAG (18:1/18:3) and SM (d18:1/20:0) (*P* < 0.01), positively correlated with the concentration of Cholesterol sulfate (*P* < 0.05). All were decreased after AEE treatment. At the Order level in Figure 12B, AEE treatment greatly decreased the abundance of Candidatus_Gastranaerophilales (*P* < 0.01). Likewise, AEE treatment decreased the abundance of Verrucomicrobiales (*P* < 0.05). The results of the joint analysis showed that compared with the HFD group, in the AEE group the abundance of Verrucomicrobiales was significantly positively correlated with the concentration of SM (d18:1/18:0) (*P* < 0.001), positively correlated with the concentrations of SM (d18:1/20:0), DAG (18:1/18:3), and PE (16:0/18:1) (*P* < 0.01), positively correlated with the concentration of Cholesterol sulfate (*P* < 0.05). Likewise, in the AEE group the abundance of Candidatus_Gastranaerophilales was significantly positively correlated with the concentration of SM (d18:1/18:0) and PE (16:0/18:1) (*P* < 0.001), positively correlated with the concentrations of SM (d18:1/20:0) and DAG (18:1/18:3), positively correlated with the concentration of Cholesterol sulfate (*P* < 0.05). At the Species level in Figure 12C, AEE treatment significantly elevated the abundance of Muribaculaceae_bacterium_Isolate-004_(NCI) (*P* < 0.01) and dramatically decreased the abundance of Clostridia_bacterium (*P* < 0.01) in comparison with HFD group. The results of the joint analysis showed that compared with the HFD group, in the AEE group the abundance of Clostridia_bacterium was positively correlated with the concentration of SM (d18:1/20:0) (*P* < 0.01), positively correlated with the concentration of SM (d18:1/18:0) (*P* < 0.05). However, in the AEE group the abundance of Muribaculaceae_bacterium_Isolate-004_(NCI) was negatively correlated with the concentration of SM (d18:1/20:0) (*P* < 0.01), was positively correlated with the concentrations of SM (d18:1/18:0), PE (16:0/18:1), and Cholesterol sulfate (*P* < 0.05).

## Discussion

The harm of cardiovascular and cerebrovascular diseases caused by hyperlipidemia is still increasing. Hyperlipidemia is caused by abnormal lipid metabolism and is mainly manifested as blood lipids disorder, the abnormal levels of TG, TCH, LDL, and HDL (19). Previous studies showed that AEE has a good effect in improving the lipid disorders in hyperlipidemia rats via decreasing the blood lipid levels of TG, TC, and LDL (10). As shown in the previous results, AEE also showed good efficacy in improving blood lipid levels of hyperlipidemia mice induced by HFD diet in this study, mainly by reducing the levels of TCH and LDL-C, and further proved that AEE had good efficacy in alleviating hyperlipidemia caused by lipid disorders. With the continuous development of lipidomics and metagenomics, more and more studies have shown that lipids and gut microbiota play important roles in the occurrence and development of hyperlipidemia (2, 17). Therefore, in order to explore the effects of lipids and gut microbiota on the efficacy of AEE in alleviating hyperlipidemia in mice, we performed lipidomics on liver and metagenomics on cecal contents.

Lipid metabolism disorder is a hallmark of various metabolic disorders, which will lead to low efficiency of lipid metabolism, thus inducing the development of obesity and dyslipidemia (20, 21). The liver is an important organ for energy metabolism and maintains lipid homeostasis by regulating the synthesis and catabolism of lipids such as free fatty acids, TG, and cholesterol. Lipid metabolism disorder in the liver is an important cause of hepatic steatosis (22). Abnormal lipid metabolism induces hyperlipidemia. Therefore, we applied lipidomics to provide new insights into the molecular mechanism of AEE in alleviating hyperlipidemia in mice in this study.

Lipid metabolism abnormalities are the most common in hyperlipidemia. In this study, abnormal lipid metabolism was manifested as the up-regulation of LPCs, PEs, PCs, SMs, and LPGs, and downregulated lipids were focused on TAGs, Cers, DAGs, PEs, and PGs in the HFD group. AEE treatment significantly down-regulated the concentrations of 12 SMs and 10 PEs. Hence, these lipids were likely to be potential biomarkers for AEE to regulate lipid metabolism in mice. According to a previous study, tangeretin decreased the SMs and PEs up-regulated by high-fat diet in rat (23). In ApoE−/− mice, the concentration of PEs significantly increased (24). Previous studies have suggested that ApoE deficiency may lead to a decrease in PSs species (24), and this result is consistent with this study. PEs belongs to glycerophospholipids, and SMs belongs to sphingolipids. SMs is an important candidate for hepatic lipid accumulation in high-fat and high-cholesterol mice by up-regulation the expression of sphingomyelin synthase1 (25). Therefore, the 12 SMs and 10 PEs may be closely related to the alleviation of hyperlipidemia in mice by AEE via sphingolipid signaling pathway and glycerophospholipid metabolism.

In liver lipidomics, AEE treatment ameliorated the pathways destroyed in the model group of choline metabolism in cancer, glycerophospholipid metabolism, GnRH signaling pathway, fat digestion and absorption, sphingolipid signaling pathway, cAMP signaling pathway, NF-kB signaling pathway, Th1 and Th2 cell differentiation. The result demonstrated that AEE participates in multiple metabolic pathways in the alleviation of hyperlipidemia in mice. This result is similar to that of many related studies (23, 26). Studies have shown that these pathways are partially abnormal in hyperlipidemia, atherosclerosis, and diseases associated with abnormal lipid metabolism (23).

Lysophosphatidylcholine (LPC) plays an important role in lipid metabolism disorders, and it mainly acts through glycerophospholipid metabolism. Glycerophospholipid metabolism plays key roles in platelet aggregation, inflammatory diseases, and hyperlipidemia development (27). In this study, these abnormal lipids were significantly enriched in glycerophospholipid metabolisms. Meanwhile, it was found that the type and concentration of glycerophospholipids in mouse liver were higher than those of other lipids. Previous studies showed that AEE reduces the level of elevated LysoPC (18: 0), LysoPC (16: 1), and LysoPC (20: 3) in the liver of hyperlipidemia hamster (10). This result is consistent with the results of this study. Moreover, we found that AEE significantly increased the level of PA (16:0/18:2) and decreased the level of PC (16:1/16:1), LPCs, PS (18:1/20:0), PE (16:1/22:4) in this study in Figure 4B. Besides LPC, AEE also regulates glycerol phospholipid metabolism through PA, PE, and DAG. Hence, AEE played a key role in regulating the disorders of lipids by glycerophospholipid metabolism, which could repress the progress of hyperlipidemia.

Sphingolipid signaling pathway is associated with fat accumulation (28). Inhibiting glycosphingolipid biosynthesis decreased atherosclerosis by inhibiting several enzymes involved in sphingolipid synthesis (29). And inhibition of sphingolipid metabolism can improve the body circulating lipids by decrease of LDL (30). In this study, AEE decreased the concentration of 12 SMs belonging to sphingolipids. Therefore, regulation of sphingolipid metabolism may be the main pathway for AEE to achieve the alleviation of hyperlipidemia in mice.

NF-kB is a key signaling pathway for inflammatory response. Excessive lipid accumulation can cause hepatic lipid toxicity (31). Thereby cause liver damage and inflammation. The function of insulin resistance is improved in the liver when inflammation and lipid accumulation are reduced (32). Many studies have shown that hyperlipidemia is accompanied by a significant increase in inflammation in the body, so inflammation is crucial in hyperlipidemia (33, 34). Previous studies have shown that AEE significantly down-regulates the inflammatory factor IL-1, TNF-alpha, and IL-6 (35). Thus NF-kB signaling pathway plays an important role in the effect of AEE on hyperlipidemia.

Gut microbiota is closely related to host health. In this study, the results of species level analysis showed that HFD induction caused gut microbiota imbalance in mice, but the effect of AEE alleviated multiple imbalance flora. One study showed that AEE decreased the abundance of Firmicutes at the Phylum level in an animal model of hyperlipidemia (36). In the current study, at the Phylum level, AEE also decreased the abundance of Firmicutes which is a second in abundance. *Huazhi Rougan granule* (HRG) can reduce lipids and protect the liver and one study showed that HRG decreased the abundance of Firmicutes in mice fed with HFD (37), which was consistent with the result of AEE. Lactobacillus Paragasseri Y20 can decrease the cholesterol level and effect the gut microbiota on rats with high cholesterol diet. Study showed that Lactobacillus Paragasseri Y20 decreased the abundance of Verrucomicrobia in the process of relieving high cholesterol rats (38), which was the same to AEE. AEE increased the abundance of Deferribacteres which changed significantly in the AEE group. AEE reduced the abundance of Tenericutes which was the main Phylum in the intestinal contents. Astragalus polysaccharides combined with berberine can reduce HFD-induced obesity and modulate the gut microbiota in mice. Astragalus polysaccharides significantly reduced the abundance of Deferribacteres (39), which was in contrast to the effect of AEE.

In this study, to find out the biomarker microbiota with statistical abundance differences between the different groups, the results of LEfSe analysis showed that Muribaculaceae, Duncaniella, and Muribaculum were species with relatively high abundance in the AEE group. These species at five taxonomic levels (from Class to Species), from C__Deferribacteres to S__*Mucispirillum_schaedleri*, from C__Flavobacteriia to S__*Capnocytophaga_felis*, and from C__Actinobacteria to S__*Bifidobacterium_adolescentis*, were significantly different species in the AEE group. Therefore, these microbiotas are likely to be the biomarker flora in AEE group. Notably, almost all species with significant changes in the AEE group were members of the Bacteroidetes (78%) and a small portion of the Firmicutes (12%). Thus, we suggested that AEE mainly improved the hyperlipidemia by changing the flora of Bacteroidetes and Firmicutes.

In the current study, functional annotation results showed that species/genes from the three groups were involved in the functions of lipid transport and metabolism, immune disease, lipid metabolism, energy metabolism, glycoside hydrolase, glycosyltransferase, carbohydrate esterases, and so on. The results of abundance information of the functions showed that compared to the HFD group, in the AEE group the significant difference functions were lipid transport, the biosynthesis of lipid A, bile acid, DNA primase activity, biosynthesis of unsaturated fatty acids, Glycosphingolipid biosynthesis, insulin signaling pathway, inositol phosphate metabolism, secondary bile acid biosynthesis, Valine, leucine and isoleucine biosynthesis, and so on. These results further indicated that AEE in alleviating hyperlipidemia was mainly involved in the functions of lipid transport and metabolism, as well as the metabolism of bile acids and secondary bile acids. This result is consistent with the previous one (10).

The process of hyperlipidemia is accompanied by lipid metabolism disorders. Therefore, the alleviation of hyperlipidemia in mice by AEE was accompanied by the regulation of lipid transport. Lipoprotein A is mainly synthesized in the liver and promotes the formation of atherosclerosis. It is an independent risk factor for stroke and coronary heart disease. In the liver, cholesterol is converted into primary bile acids, which are then excreted in the feces. This process is the key way to reduce the accumulation of cholesterol in the body. BAs play a vital role in the regulation of lipid metabolism (40). Therefore, the regulation of bile acids is essential for the relief of hyperlipidemia. Studies have shown that sterile mice have no secondary bile acids (41). Therefore, the protective effect of gut microbiota on hyperlipidemia is likely caused by the regulation of secondary bile acids biosynthesis. Thus, bile acid regulation and secondary bile acid biosynthesis play a dominant role in the alleviation of hyperlipidemia in mice by AEE. One study demonstrates that the biosynthesis of unsaturated fatty acids is closely related to host lipid metabolism (42). The results showed that AEE reduced the biosynthesis of unsaturated fatty acids in hyperlipidemia mice, which may be due to the compensatory reaction. A mouse study showed that inhibiting glycosphingolipid biosynthesis decreased atherosclerosis (29). In this study, AEE treatment markedly decreased the function abundance of glycosphingolipid biosynthesis, suggesting that AEE relieves hyperlipidemia in mice by regulating the glycosphingolipid biosynthesis. Liver lipids were found to be negatively correlated with insulin sensitivity. The accumulation of hepatic triglyceride, however, may reduce insulin clearance and result in peripheral insulin resistance (43, 44). Insulin resistance is a major contributing factor to fatty liver disease. And one study suggests that impaired lipogenesis and increased lipid infiltration in the liver occurred in the early stage of the onset of insulin resistance (45). In this study, AEE treatment significantly down-regulated the biosynthesis of valine, leucine, and isoleucine. One study suggests that the branched chain amino acids isoleucine, leucine, valine, may play a role in the development of insulin resistance and diabetes, and have been used as predictive indices of diabetes development (46). Our results indicated that hyperlipidemia in mice is accompanied by changes in insulin regulation and Valine, leucine, and isoleucine biosynthesis. Hence, the alleviation of hyperlipidemia in mice by AEE was accompanied by the regulation of insulin resistance by valine, leucine, and isoleucine. These results indicated that AEE might regulate the gut microbiota of hyperlipidemia mice and alleviate hyperlipidemia through the above pathways.

The sterile ApoE−/− mice treated with HFD had more severe hyperlipidemia and more atherosclerotic plaques than normal ApoE−/− mice (47). This result indicated that the gut microbiota had a protective effect on the progression of hyperlipidemia in mice. Cholesterol is converted to primary bile acids in the liver and primary bile acids are converted to secondary bile acids by the intestinal flora. Studies have shown that sterile mice have a larger gall bladder and more primary bile acids, and no secondary bile acids (41). Therefore, we speculate that the protective effect of intestinal flora on hyperlipidemia is likely caused by affecting the bile acid metabolism of the body, especially involving in the conversion of primary bile acids into secondary bile acids. FXR is a nuclear receptor that regulates lipid and glucose metabolism, and bile acids are activators of FXR receptors. Many studies showed that FXR inhibits CYP7A1 a key enzyme in the liver for the conversion of cholesterol to primary bile acids (48, 49). Previous studies have shown that AEE has a significant effect on bile acids content in rat liver, mainly through the down-regulation of FXR expression and up-regulation of CYP7A1 expression. Therefore, we speculated that AEE probably affected the metabolism of bile acids by regulating the gut microbiota, resulting in the down-regulation of FXR expression and up-regulation of CYP7A1 expression to alleviate hyperlipidemia in the body.

The gut microbiota plays an important role in diseases caused by lipid metabolism disorders, such as atherosclerosis and hyperlipidemia, mainly via affecting lipid and bile acid metabolism (50, 51). Cholesterol transport is an important component of lipid metabolism (52). To further elucidate the correlation between lipids and gut microbiota in the response of AEE to hyperlipidemia in mice, we performed a joint analysis of the hepatic lipidomics and the metagenome of the cecal contents. In the AEE group the abundance of Verrucomicrobia, Verrucomicrobiales, Candidatus_Gastranaerophilales, and Candidatus_Melainabacteria was significantly positively correlated with the concentration of SM (d18:1/18:0) and PE (16:0/18:1). These results suggested that the biomarkers of AEE alleviating hyperlipidemia in mice were likely to be species/genes of Candidatus_Melainabacteria, Verrucomicrobia, Candidatus_Gastranaerophilales, Verrucomicrobiales, and the metabolites of SM (d18:1/18:0), PE (16:0/18:1).

## Conclusion

AEE treatment could effectively alleviate hyperlipidemia in mice by affecting gut microbiota and liver lipids. Lipidomics analysis showed that these beneficial effects of AEE in hyperlipidemia mice were associated with the downregulation of PEs and SMs in the liver, and were mediated mainly through the glycerophospholipid metabolic pathway, sphingolipid signaling pathway, and NF-kB signaling pathway. Metagenomics showed that these beneficial effects of AEE were associated with up-regulations of deferribacteres, deferribacterales, bacteroidales_bacterium and muribaculaceae_bacterium, and down-regulations of firmicutes, verrucomicrobia, Verrucomicrobiales, Candidatus_Melainabacteria and candidatus_gastranaerophilales in gut microbiota, and were primarily mediated by bile acid metabolism. The joint analysis further illustrates that these beneficial effects are likely due to the abundance of Verrucomicrobia, Verrucomicrobiales, Candidatus_Gastranaerophilales, and Candidatus_Melainabacteria significantly positively correlated with the concentration of SM (d18:1/18:0) and PE (16:0/18:1) in the AEE group. It was also demonstrated that the combination of lipidomics and metagenomics approach was a powerful tool in investigating drug action mechanism.

## Data availability statement

The data presented in this study has been deposited in the Genome Sequence Archive (GSA) repository, accession number CRA009165 (https://ngdc.cncb.ac.cn/gsa/browse/CRA009165).

## Ethics statement

The protocols and procedures for the animal study were approved by the Institutional Animal Care and Use Committee of Lanzhou Institute of Husbandry and Pharmaceutical Science of Chinese Academy of Agricultural Sciences (Approval No. NKMYD202108; Approval Date: 20 May 2021). Animal welfare and experimental procedures were performed strictly in accordance with the Guidelines for the Care and Use of Laboratory Animals issued by the US National Institutes of Health.

## Author contributions

J-YL designed the experiments and wrote the manuscript. X-RL designed and performed the experiments and wrote the manuscript. Y-JY designed the experiments and synthesized AEE. S-HL, L-XB, X-WL, ZQ, and W-BG supplied reagents. All authors contributed to the article and approved the submitted version.
