# Supplementary material for: Untargeted lipidomics and metagenomics reveal the mechanism of aspirin eugenol ester relieving hyperlipidemia in ApoE−/− mice
Source: Front Nutr. 2022 Dec 19;9:1030528. doi: 10.3389/fnut.2022.1030528 (PMC9815714; doi:10.3389/fnut.2022.1030528)
Supplement: Supplementary file 1 [file Data_Sheet_1.docx]

**Fig. S1** The body weight of mice in each group.

**Fig. S2** TIC in both positive and negative modes. Sample QC-1: First QC sample. There were four QC samples in total for this study. (A) TIC in positive modes. (B) TIC in negative modes.

**
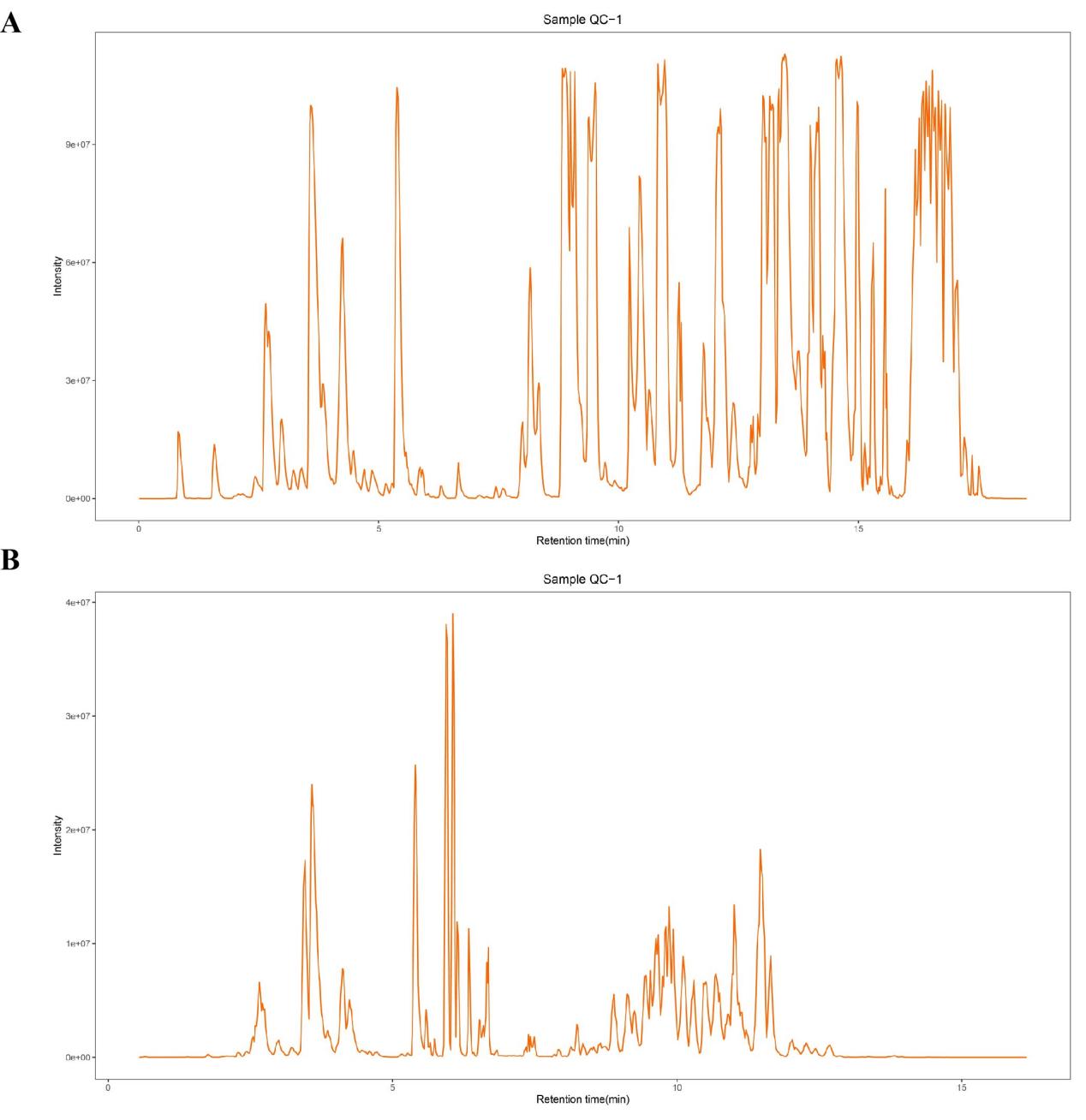
**

**Fig. S3** PCA and PLS-DA analysis of lipid in the liver.


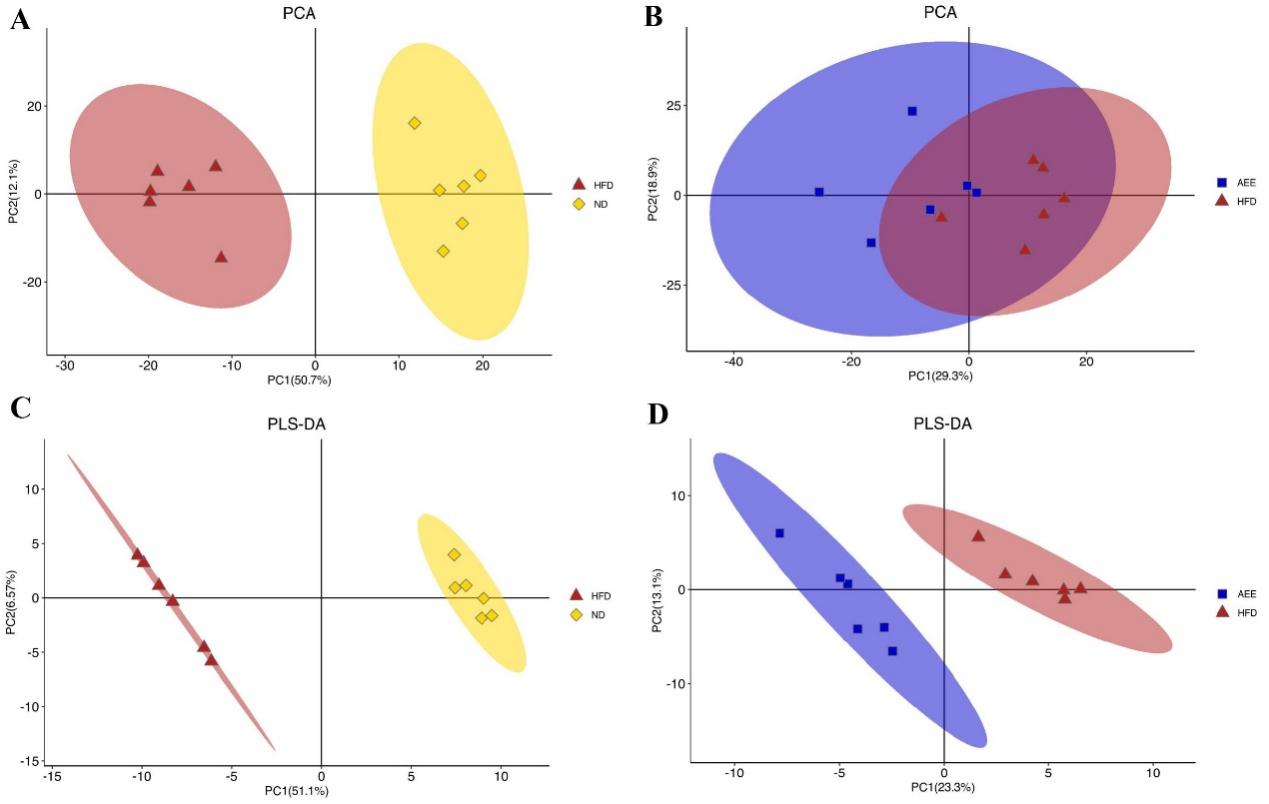


(A) PCA analysis in HFD vs ND. (B) PCA analysis in AEE vs HFD. (C) PLS-DA analysis in HFD vs ND. (D) PLS-DA analysis in AEE vs HFD.

**Fig. S4** Gene level analysis of metagenomic sequence. (A) The Core and Pan genes dilution curves. The abscissa represents the number of samples extracted; The ordinate represents the number of Core_Pan genes of the extracted sample combination. (B) Calculation formula of gene abundance. R represents the number of reads of the compared gene; L represents the length of a gene. (C) Venn diagram analysis of gene number. The number in that Core represent the number of gene common to all samples, and the numbers on the petals represent the total numb of genes of each sample minus the number of common genes. (D) Violin figure of gene number between groups. The horizontal coordinate was used for grouping, and different groups were distinguished by different colors. The vertical coordinate was used for gene number, and ns was considered as no difference.

**
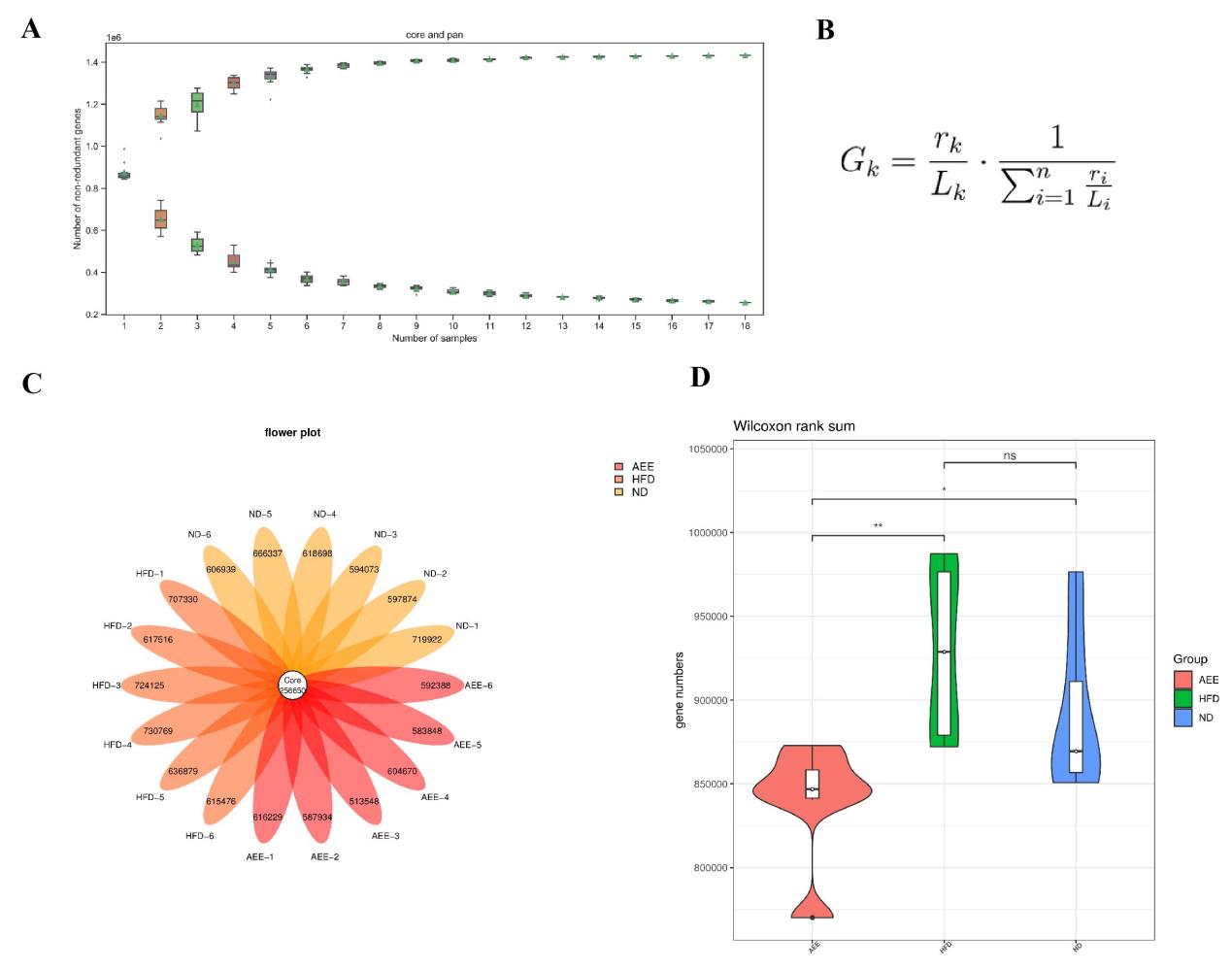
**

**Fig. S5** Number of annotation genes for multiple databases.


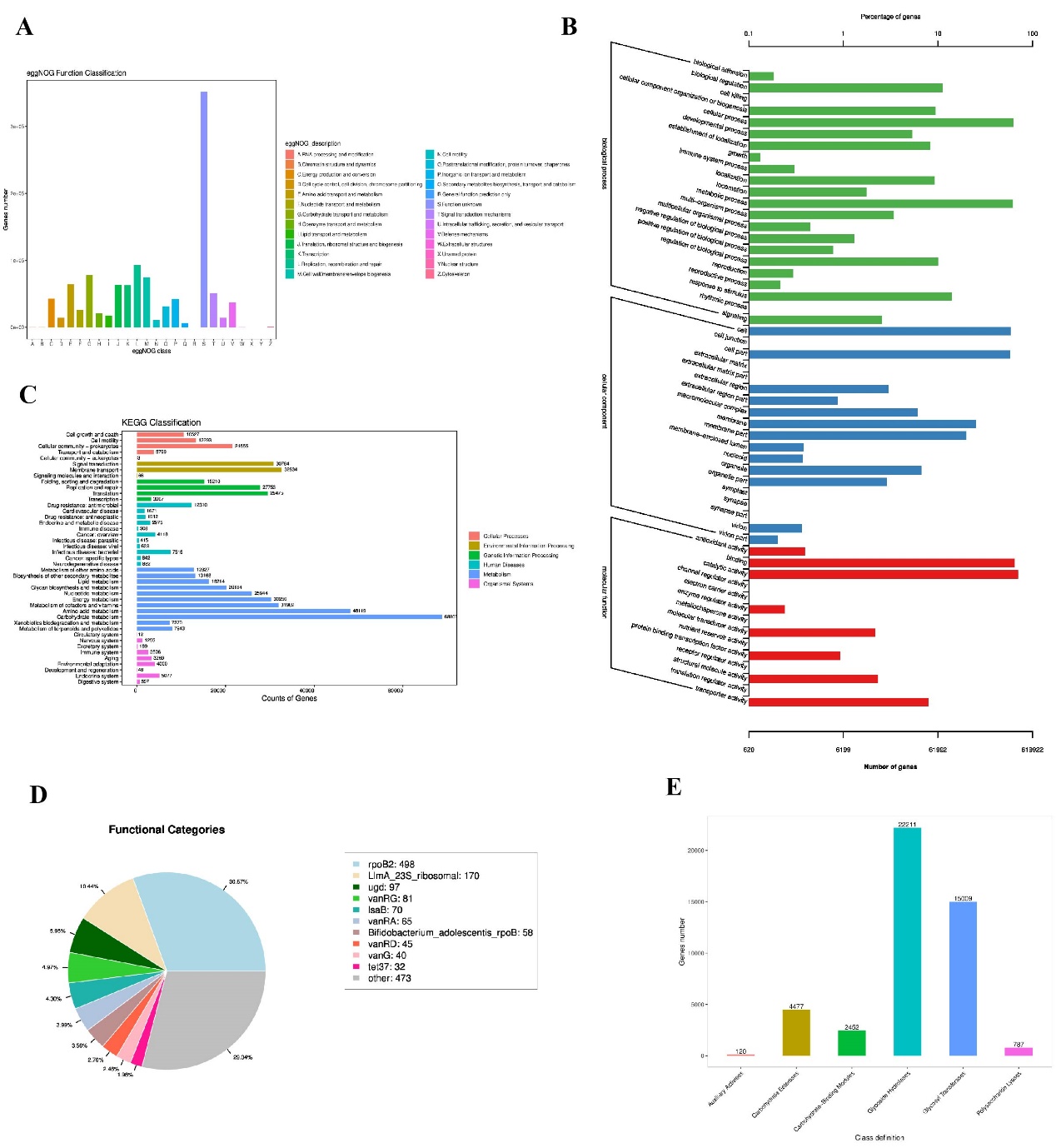


Statistical diagram of annotated gene number in eggNOG database (A), KEGG (C) and CAZy (E). (B) GO functional classification diagram. (D) CARD database annotation top10 ARO presentation figure.

**Table S1** The elution gradient.

| Time（min） | Solvent. B（%） |
| --- | --- |
| 0.0 | 0 |
| 1.5 | 0 |
| 5.0 | 55 |
| 10.0 | 60 |
| 13.0 | 70 |
| 15.0 | 90 |
| 16.0 | 100 |
| 18.0 | 100 |
| 18.1  20.0 | 0 |
| 20.0 | 0 |
